# Controlling the angle between the distal locking screw and tibiotalar joint tangent helps to reduce the occurrence of misalignment of distal tibial fractures treated with intramedullary nail fixation

**DOI:** 10.1186/s12891-022-05641-x

**Published:** 2022-07-14

**Authors:** Miao He, Jian Liu, Xu Deng, Miao He

**Affiliations:** Department of Orthopaedic Surgery, Chongqing Emergency Medical Center (Chongqing University Central Hospital), No. 1 Jiankang Road, Chongqing, 400010 China

**Keywords:** Intramedullary nail, Tibial fracture, Misalignment

## Abstract

**Background:**

Studies have shown that on the coronal plane, whether the direction of the distal locking screw is parallel to the tangent line of the tibiotalar joint can be used to determine whether there is varus or valgus deformity after the treatment of distal tibial fractures with intramedullary nail (IMN) fixation. However, there has been no statistical analysis of the included angle on the coronal plane, and there have been no reports on whether there is a relationship between the direction of the distal locking screw on the sagittal plane or the included angle of the tangent line of the tibiotalar joint and the postoperative alignment of distal tibial fractures treated with IMN fixation.

**Objective:**

Our aim was to evaluate the relationship between the angles formed by the distal locking screw and the tibiotalar joint tangent (ADTTs) on the sagittal and coronal planes and postoperative alignment in the treatment of distal tibial fractures with IMN fixation.

**Methods:**

We performed a retrospective analysis of 100 patients with distal tibial fractures treated with IMN fixation using the suprapatellar approach. On the coronal and sagittal planes, the ADTTs were arranged from small to large and divided into 4 groups, namely, groups A, B, C and D. One-way ANOVA was used to compare the lateral distal tibial angle (LDTA) and anterior distal tibial angle (ADTA) among all groups, and the chi-square test was used to compare the incidence of postoperative tibial misalignment among all groups. Univariate analysis was performed using chi-square tests to identify factors that might be associated with dislocation, including fibular open reduction and internal fixation (ORIF), limited open reduction, ADTT, IMN diameter, injury mechanism, open vs. closed fracture, comminution, and fibular fracture level. Then, the statistically significant variables in the univariate analysis were included in a multivariate logistic regression equation to evaluate the independent factors related to misalignment.

**Results:**

On the coronal plane, the ADTTs of groups A, B, C and D were < 0°, 0°-1.3°, 1.3°-2.7° and > 2.7°, respectively. The mean LDTAs of groups B and C (0°-1.3° and 1.3°-2.7°), group A (< 0°) and group D (> 2.7°) were 89.5 ± 1.6°, 92.0 ± 3.2° and 85.8 ± 3.5°, respectively (*P* < 0.01). Deformity greater than 5° were more likely in groups A and D than groups B and C [14 of 50 (28%) vs. 1 of 50 (2%), *P* < 0.001]. On the sagittal plane, the ADTTs of groups A, B, C and D were < 8.9°, 8.9°-10.4°, 10.4°-11.7° and > 11.8°, respectively. The average ADTAs of groups B and C (8.9°-10.4° and 10.4°-11.7°), group A (< 8.9°) and group D (> 11.8°) were 80.4 ± 1.3°, 83.1 ± 3.7° and 77.9 ± 2.5°, respectively (*P* < 0.01). Deformity greater than 5° was more likely in groups A and D than groups B and C [13 of 50 (26%) vs. 0 of 50 (0%), *P* < 0.001]. An ADTT of 0°-2.7° on the coronal plane and 8.9°-11.7° on the sagittal plane (OR: 0.08, *P* = 0.02) and limited open reduction (OR: 0.21, *P* < 0.01) were independent factors that reduced the likelihood of misalignment.

**Conclusion:**

The alignment of distal tibial fractures after surgery is sensitive to the ADTT and use of limited open reduction. Controlling the ADTT between 0° and 2.7° on the coronal plane and between 8.9° and 11.7° on the sagittal plane is helpful to reduce the occurrence of misalignment after the treatment of distal tibial fractures by IMN fixation.

## Background

Tibial intramedullary nail (IMN) fixation has become the standard treatment for displaced tibial fractures in adults due to its low interference with the soft tissue surrounding the fracture site and high rate of fracture healing [1–3]. Meanwhile, IMN fixation has been proven to be an effective method for the treatment of distal tibial fractures; even when the fracture line involves the joint, IMN-assisted screw fixation can be used to manage this type of fracture [4]. At present, tibial IMN fixation is divided into infrapatellar and suprapatellar approaches. The suprapatellar approach has many advantages [5–7] and allows distal tibial fractures to be reduced better and more easily [5, 8].

Due to the widening and filling of the distal tibial canal with weak cancellous bone [9], IMNs can be inserted into the distal canal from multiple angles. It has been reported that approximately 5–30% of IMN fixation procedures for distal tibial fractures result in misalignment [4, 10–22]. In addition, due to the closed reduction of the fractured end, the fractured end cannot be directly examined. Intraoperative C-arm fluoroscopy can be used to observe the fracture site only rather than the entire tibia, which makes it particularly difficult to control misalignment. It has also been reported that even a small amount of residual misalignment alters the loading of the knee and ankle joints [23], and the alteration in the force distribution may contribute to a predisposition to osteoarthritis [17].

Whether distal tibial fractures are misaligned when they are fixed with IMNs is determined intuitively during surgery. Our objective was to evaluate the relationship between the ADTT and postoperative alignment on the sagittal and coronal planes when treating distal tibial fractures with IMN fixation using the suprapatellar approach. Our hypothesis was that the ADTT is related to the postoperative alignment of distal tibial fractures.

## Materials and methods

### Study design

We performed a retrospective analysis of all patients with distal tibial fractures treated with IMN fixation from January 2018 to March 2022. We defined distal-third tibial fractures by an extension of the primary fracture line within 11 cm of the plafond, as previously described [18]. We initially enrolled 134 patients, excluding 16 with inadequate postoperative imaging data, 11 treated with plate-assisted tibial shaft fixation, and 7 treated without distal anterior-posterior locking screws. Finally, 100 study patients (117 OTA/AO fracture of dislocation classification type 42 and 13 OTA/AO fractures of dislocation type 43) were included [23].

Thirty-six open fractures were treated with irrigation and debridement before final fixation. Fibular fractures occurred in 85 patients, and limited open reduction was performed before tibial IMN fixation in 47 patients. Eight fractures included nondisplaced or slightly displaced intra-articular fractures that were fixed with compression screws prior to IMN fixation. All fractures were treated with suprapatellar IMN fixation and specialized tibial nailing.

### Digital radiography (DR) measurements

Our facility protocol is to review the full-length tibial X-ray before extubation. Alignment was assessed by measuring the lateral distal tibial angle (LDTA) and the anterior distal tibial angle (ADTA). Normal absolute coronal and sagittal alignment were defined as an LDTA of 89° and an ADTA of 80°, respectively, in accordance with Paley [24]. Deviations from these measurements of more than 5° were defined as coronal and sagittal misalignment, respectively.

**Fig. 1:**
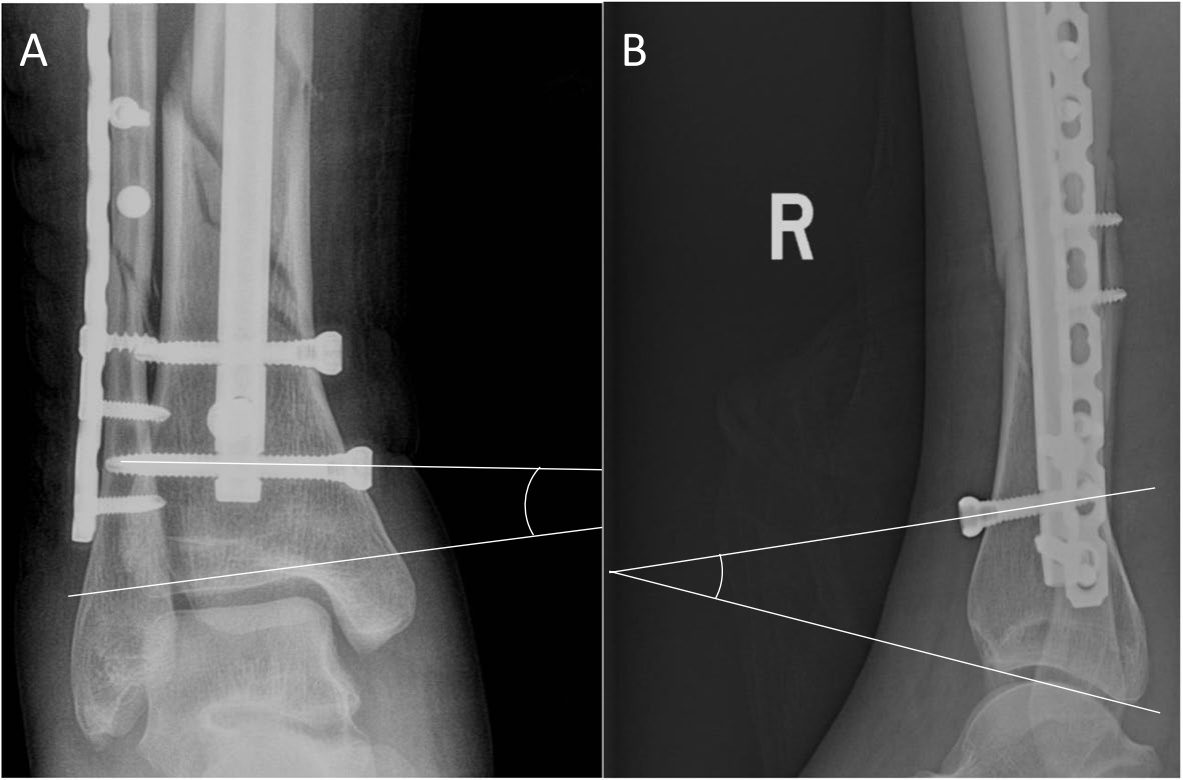
Measurement of the ADTT. **A** On the coronal plane, the ADTT was measured between the projection line of the distal interlocking screw in the mediolateral direction and the tibiotalar joint tangent. The angle was negative when pointing medially. **B** On the sagittal plane, the ADTT was measured between the projection line of the distal interlocking screw in the anteroposterior direction and the tibiotalar joint tangent. The angle was positive when pointing in the anterior direction

The ADTT was measured between the projection line of the distal interlocking screw in the mediolateral direction and the tibiotalar joint tangent on the coronal plane. The ADTT was positive when pointing in the lateral direction and negative when pointing in the medial direction. The ADTT was measured between the projection line of the distal interlocking screw in the anteroposterior direction and the tibiotalar joint tangent on the sagittal plane. If the angle pointed in the anterior direction, the ADTT was positive; if it pointed in the posterior direction, the ADTT was negative (Fig. 1). All measurements for the whole cohort were made by 2 blinded reviewers (Liu J, Deng X) to ensure the reliability of the reviewers. To assess intraobserver reliability, one reader (Liu J) repeated all measurements 1 month later.

### Statistical analysis

IBM SPSS 20.0 (IBM Corp. New York, USA) was used for the statistical analyses. The significance level was set as 0.05. Quantitative data are represented as the mean ± standard deviation. The angles between the distal screws and the tibiotalar plane on the coronal and sagittal planes were arranged from small to large and divided into 4 groups. The ADTA and LDTA of each group were compared by one-way ANOVA, and the incidence of malformation was evaluated and compared by chi-square analysis. Univariate analysis was performed using chi-square tests to identify factors that might be associated with dislocation, including fibular open reduction and internal fixation (ORIF), limited open reduction, ADTT, IMN diameter, injury mechanism, open vs. closed fracture, comminution, and fibular fracture level. Then, the statistically significant variables in the univariate analysis were included in a multivariate logistic regression equation to evaluate the independent factors related to misalignment.

### Ethics approval

This retrospective study involving human participants was conducted in accordance with the ethical standards of the institutional and national research committee and with the 1964 Helsinki Declaration and its later amendments or comparable ethical standards. The study was approved by the Ethics Committee of the Central Hospital affiliated with Chongqing University. Informed consent was obtained from all participants.

## Results

One hundred patients, consisting of 23 females and 77 males, with an average age of 44.72 ± 15.14 years, were included in the final analysis. The intra- and interobserver reliability of the LDTA was 0.84 (95% CI, 0.77–0.89) and 0.83 (95% CI, 0.75–0.89), respectively. The intra- and interobserver reliability of the ADTA was 0.82 (95% CI, 0.75–0.89) and 0.81 (95% CI, 0.71–0.87), respectively. The intra- and interobserver reliability of the ADTT on the coronal plane was 0.83 (95% CI, 0.76–0.88) and 0.89 (95% CI, 0.83–0.93), respectively. The intra- and interobserver reliability of the ADTT on the sagittal plane was 0.81 (95% CI, 0.73–0.87) and 0.87 (95% CI, 0.81–0.92), respectively (Table 1).

**Table 1:** Inter- and intraobserver reliability of all DR measurements^a^

	Intraobserver reliability	Interobserver reliability
LDTA	0.84 (0.77–0.89)	0.83 (0.75–0.89)
ADTA	0.82 (0.75–0.89)	0.81 (0.71–0.87)
ADTT (coronal plane)	0.83 (0.76–0.88)	0.89 (0.83–0.93)
ADTT (sagittal plane)	0.81 (0.73–0.87)	0.87 (0.81–0.92)

^a^Values are presented as the intraclass correlation (95% CI)

*LDTA* Lateral distal tibial angle, *ADTA* Anterior distal tibial angle, *ADTT* Angle formed by distal locking screw and tibiotalar joint tangent

We found 23 cases (23%) of angular deformity greater than 5° on any plane. Fifteen cases (15%) showed abnormal coronal plane alignment: 8 cases were valgus, with 1 case (1%) of valgus deformity greater than 10°; and 7 cases were varus, with 1 case (1%) of varus deformity greater than 10°. There were 13 cases of sagittal deformity (13%), including 6 cases of procurvatum deformity (6%) and 7 cases of recurvatum deformity (7%); of these, there were 2 cases of recurvatum deformity (2%) greater than 10°. There were 5 cases (5%) of deformity on both the coronal and sagittal planes, including 2 cases of valgus and recurvatum (2%), 2 cases of valgus and procurvatum (2%) and 1 case of varus and recurvatum (1%).

**Fig. 2:**
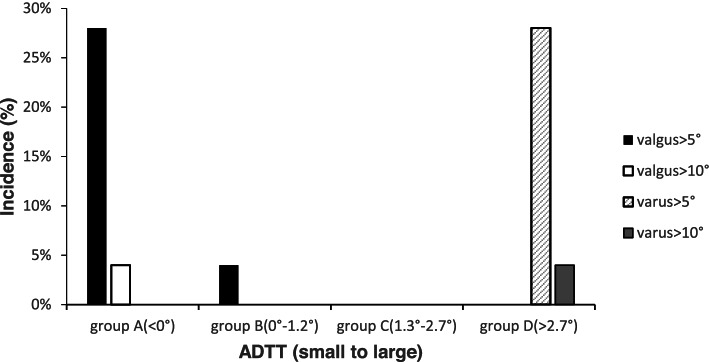
Coronal plane: significant difference in malalignment between groups A and D and groups B and C

The ADTTs (coronal plane) were arranged from small to large and divided into 4 groups, namely, groups A, B, C and D, with 25 cases in each group. The ADTTs of groups A, B, C and D were < 0°, 0°-1.3°, 1.3°-2.7° and > 2.7°, respectively. The valgus deformity rate was 28% in group A (*n* = 7/25, mean LDTA = 92.0 ± 3.2°), and all fractures with valgus deformity greater than 10° were in this group (1 of 25, 4%). The varus deformity rate in group D was 28% (*n* = 7/25, mean LDTA = 85.8 ± 3.5°), and all fractures with varus deformity greater than 10° were in this group (1 of 25, 4%) (Fig. 2). Compared with groups B and C (0°-1.3° and 1.3°-2.7°), group A (< 0°) presented relative valgus, and group D (2.7°) presented relative varus (mean LDTA: 89.5 ± 1.6° vs. 92.0 ± 3.2° vs. 85.8 ± 3.5°, *P* < 0.01). Misalignment greater than 5° was more likely in groups A and D than groups B and C [14 of 50 (28%) vs. 1 of 50 (2%), *P* < 0.001].

**Fig. 3:**
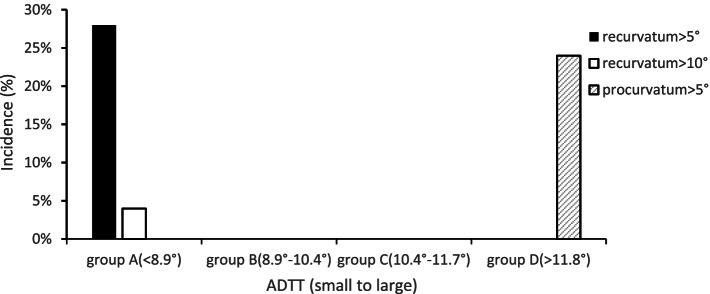
Sagittal plane: significant difference in malalignment between groups A and D and groups B and C

Similarly, the ADTTs (sagittal plane) were arranged from small to large and divided into 4 groups, namely, groups A, B, C and D, with 25 cases in each group. The ADTTs of groups A, B, C and D were < 8.9°, 8.9°-10.4°, 10.4°-11.7° and > 11.8°, respectively. The recurvatum deformity rate was 28% in group A (*n* = 7/25, mean ADTA = 83.1 ± 3.7°). All fractures with recurvatum deformity greater than 10° were in this group (2 of 25, 8%). In group D, the rate of procurvatum deformity was 24% (*n* = 6/25, mean ADTA = 77.9 ± 2.5°) (Fig. 3). Compared with patients in groups B and C (8.9°-10.4° and 10.4°-11.7°), patients in group A (< 8.9°) presented relative recurvatum, and patients in group D (> 11.8°) presented relative procurvatum (mean ADTA: 80.4 ± 1.3° vs. 83.1 ± 3.7° vs. 77.9 ± 2.5°, *P* < 0.01). Misalignment greater than 5° was more likely in groups A and D than groups B and C [13 of 50 (26%) vs. 0 of 50 (0%), *P* < 0.001].

The results of the univariate analysis of treatment and injury variables are shown in Table 2. An ADTT on the coronal plane of 0°-2.7° and an ADTT on the sagittal plane of 8.9°-11.7° resulted in significantly lower incidences of misalignment greater than 5° than other angles [1/28 (4%) vs. 22/72 (31%), *P* < 0.01]. The probability of 5° of misalignment was significantly lower in the limited open reduction group than in the other groups [8/47 (17.0%) vs. 15/53 (28.3%), *P* = 0.02]. Fibular ORIF, IMN diameter, mechanism of injury, open vs. closed, comminution, and fibular fracture at the same level were not associated with misalignment.

**Table 2:** Univariate and multivariate analyses of all factors

	Deformity> 5°	Total	Univariate analysis	Multivariate analysis		
			*p* value	*p* value	OR	95% CI
Fibular ORIF						
Yes	12 (29%)	41	0.21			
Not	11 (19%)	59				
Limited open reduction						
Yes	8 (17%)	47	0.02	< 0.01	0.21	1.15–9.84
No	15 (28%)	53				
ADTT						
Coronal plane, 0^°^-2.7^°^; Sagittal plane, 8.9^°^-11.7^°^	1 (4%)	28	< 0.01	0.02	0.08	0.01–0.65
All others	22 (31%)	72				
IMN diameter						
≥ 10	14 (21%)	67	0.48			
< 10	9 (27%)	33				
Mechanism of injury						
High energy	13 (20%)	64	0.39			
Low energy	10 (28%)	36				
Open vs. closed						
Open	4 (11%)	35	0.10			
Closed	19 (29%)	65				
Comminution						
Yes	20 (25%)	79	0.39			
No	3 (14%)	21				
Fibular fracture at same level						
Yes	9 (24%)	37	0.81			
No	14 (22%)	63				

*OR* Odds ratio, *ORIF* Open reduction and internal fixation, *ADTT* Angle formed by distal locking screw and tibiotalar joint tangent, *IMN* Intramedullary nail

The statistically significant variables in the univariate analysis were included in a multivariate logistic regression equation. An ADTT on the coronal plane of 0°-2.7° and an ADTT on the sagittal plane of 8.9°-11.7° (OR: 0.08, *P* = 0.02) and limited open reduction (OR: 0.21, *P* < 0.01) were independent factors that reduced the likelihood of misalignment (Table 2).

## Discussion

The overall incidence of misalignment on any plane (23%) is consistent with the range reported in other studies [4, 10–21]. On the coronal plane, misalignment greater than 5° was more likely in group A, with an ADTT of < 0°, and group D, with an ADTT of > 2.7°, than in groups B and C, with an ADTT of 0°-2.7° (28% vs. 0%, *P* < 0.001). On the sagittal plane, misalignment greater than 5° was more likely in group A, with an ADTT of < 8.9°, and group D, with an ADTT of > 11.8°, than groups B and C, with an ADTT of 8.9°-11.7° (26% vs. 0%, *P* < 0.001). Additionally, an ADTT on the coronal plane of 0°-2.7° and an ADTT on the sagittal plane of 8.9°-11.7° were associated with a reduced likelihood of misalignment (OR: 0.08, *P* = 0.02). In this study, the ADTT was used to evaluate the alignment of distal tibial fractures postoperatively, providing a new method to help clinicians identify whether tibial fractures were misaligned intraoperatively.

The principle of determining the presence of misalignment after distal tibial fracture fixation using the ADTT is as follows. In theory, when the distal tibia is anatomically reduced after surgery, the long axis of the IMN and the anatomical axis of the tibia basically overlap, and the projection line of the distal locking screw on the sagittal and coronal planes is perpendicular to the long axis of the IMN [25]. Furthermore, the included angle (LDTA and ADTA) between the anatomical axis of the tibia and the tibiotalar joint tangent is within the normal range. Therefore, the angle between the projection line of the distal interlocking screw of the tibial IMN instrumentation and the tibiotalar joint tangent on the sagittal and coronal planes also has a normal range. When the angle between the projection line of the distal interlocking screw of the tibial IMN instrumentation on the sagittal and coronal planes and the tibiotalar joint tangent is beyond the normal range, there is postoperative distal tibial fracture deformity. Lu et al. found that the tibial deformity on the coronal plane could be determined by observing whether the projection line of the distal interlocking screw of the tibial IMN instrumentation from medial to lateral was parallel to the tangent line of the tibiotalar joint [25]. However, the above study only observed whether the two lines were parallel on the coronal plane and neither carried out specific measurement and statistical analysis of the included angle nor studied the included angle on the sagittal plane.

In the multivariate analysis, we found that limited open reduction was another independent factor that reduced the likelihood of misalignment (OR: 0.21, *P* < 0.01). Previous studies have found that for distal tibial fractures, the plate shows less misalignment after limited open reduction than after IMN fixation [19, 20, 26]. The main reason is that limited open reduction can be performed during the operation and allows direct viewing of the broken end, resulting in better reduction. The results of this study are consistent with these findings.

Different methods can be used to determine the presence of misalignment after surgery for distal tibial fractures, and it is complicated to do so using the ADTT. Previous studies have compared the position of the distal nail target in the medullary cavity of the distal tibia to determine the presence of misalignment after surgery for distal tibial fractures [27, 28], and this method is simple. However, in some special cases, this method is not very suitable, and the alignment needs to be evaluated by the ADTT. When there is only accumulated metaphyseal deformity in the distal tibia before injury, the position of the distal nail target in the medullary cavity of the distal tibia cannot be determined. In this case, it is necessary to determine the presence of deformity using the ADTT.

There are also some limitations to this study. The sample size of this retrospective study is relatively small, which may increase the probability of bias in the statistical results. Therefore, future prospective studies should include larger samples to validate our findings. At the same time, the rotation of the affected limb can affect the accuracy of measurement during imaging, and we recommend further research to identify more accurate and reliable imaging techniques to improve the accuracy of measurement. This method requires at least one distal locking screw in the mediolateral direction and one locking screw in the anteroposterior direction. When misalignment is found, the locking screw must be removed and reinserted after reduction, which may cause additional damage to the distal tibia.

## Conclusion

The alignment of distal tibial fractures after surgery is sensitive to the ADTT and use of limited open reduction. The ADTT should be controlled to within 0°-2.7° on the coronal plane and 8.9°-11.7° on the sagittal plane to effectively reduce the occurrence of misalignment after IMN fixation for distal tibial fractures.

## Data Availability

The datasets generated and/or analysed during the current study are not publicly available as they contain information that could compromise the privacy of research participants but are available from the corresponding author on reasonable request.

## Abbreviations

IMN: Intramedullary nail
ADTT: Angle formed by distal locking screw and tibiotalar joint tangent
LDTA: Lateral distal tibial angle
ADTA: Anterior distal tibial angle
ORIF: Open reduction and internal fixation
OR: Odds ratio
